# Nurses' Experiences of Workplace Flourishing in Healthcare: A Qualitative Study

**DOI:** 10.1002/nur.70109

**Published:** 2026-08-01

**Authors:** Annanolli Josefiina, Mäenpää Tiia, Kuha Suvi, Kanste Outi

**Affiliations:** ^1^ Research Unit of Health Sciences and Technology University of Oulu Oulu Finland; ^2^ Medical Research Center Oulu Oulu University Hospital, University of Oulu Oulu Finland

**Keywords:** healthcare, nurse, qualitative study, work community, workplace flourishing

## Abstract

To describe nurses' experiences of workplace flourishing in healthcare. A qualitative descriptive design using content analysis. Semi‐structured remote individual interviews were conducted with 16 nurses between January and April 2023 in a private occupational healthcare organization in Finland. The data were analyzed using a deductive–inductive content analysis approach informed by the Flourishing at Work framework, which addresses the emotional, psychological, and social dimensions of workplace flourishing. Emotional well‐being included experiencing mixed work‐related emotions and energizing work performance. Psychological well‐being included fostering employee engagement, supporting professional growth, developing professional self‐awareness, recognizing the broader significance of work, and enhancing personal well‐being at work. Social well‐being included building an inclusive workplace environment, guiding actions through core values, fostering communality in the workplace, promoting mutual respect in the workplace, and experiencing human‐centered leadership. A positive work community was a central driver of all WF dimensions, highlighting its social aspect. Human‐centered leadership, characterized by coaching, empowerment, and interpersonal support, was essential for fostering WF. Nurses' WF was further shaped by personal values, meaningful work, and growth opportunities. Influence over one's work and organizational support for work–life balance also contributed significantly, underscoring the need to embed these elements in organizational practices. Findings highlight the need for human‐centered leadership and supportive work communities to enhance nurse retention and well‐being. Nurse supervisors play a key role in recognizing individual needs and supporting holistic well‐being of nurses. The reporting is based on the Consolidated Criteria for Reporting Qualitative Research (COREQ).

## 1 Introduction

The well‐being of working professionals has gained increasing attention in recent years (Peethambaran and Naim 2025), including in healthcare, where work is often characterized by psychosocial strain. Although the negative effects of burnout and psychological distress for both providers and patients are well documented, the positive well‐being of healthcare professionals remains less explored (Mascaro et al. 2022). Existing research has largely focused on illness and dysfunction, leaving gaps in understanding the conditions that support flourishing (Agenor et al. 2017).

Supporting employee flourishing has become increasingly important for organizations, as it is associated with outcomes such as work engagement (Fabricio et al. 2022) and extra‐role performance (A'yuninnisa et al. 2024). In healthcare, organizational support and social connectedness are key factors enabling professionals to flourish and sustain positive work attitudes (Bossard et al. 2024). Despite evidence linking flourishing to favorable health, behavioral, and performance outcomes (A'yuninnisa et al. 2024), on nurses' workplace flourishing (WF) remains limited, particularly regarding their subjective experiences. This study aims to describe nurses' experiences of workplace flourishing, focusing on the emotional, psychological, and social dimensions of well‐being.

## 2 Background

The concept of flourishing generally refers to positive individual and social functioning (Diener et al. 2010). In their concept analysis, Agenor et al. (2017) identified six core attributes—positive relationships, meaning and purpose, engagement, competence, positive emotions, and self‐esteem—and reviewed four theoretical models, among which Keyes (2002), encompassing emotional, psychological, and social well‐being, is the most widely validated and most comprehensively described (Agenor et al. 2017).

Recently, the concept has been extended to workplace flourishing (WF) (Peethambaran and Naim 2025; Rautenbach 2015), describing employees' experience of functioning effectively and feeling well at work (Rautenbach 2015), which encompasses emotional, psychological, and social well‐being (Peethambaran and Naim 2025). It is commonly examined using the Flourishing at Work framework (Rautenbach 2015), on which the Flourishing at Work Scale (FAWS) is based and structured according to its three dimensions. WF has been linked to organizational commitment, lower turnover intention, job satisfaction, and prosocial behavior (A'yuninnisa et al. 2024).

WF is supported by various job, organizational and individual psychological factors, including role clarity (Rautenbach 2015), work–life balance (A'yuninnisa et al. 2024; Rautenbach 2015), person–environment fit (A'yuninnisa et al. 2024), organizational support (Bossard et al. 2024), positive relationships with supervisors and colleagues (Muñiz‐Velázquez et al. 2022), and individual resources such as self‐esteem, cultural intelligence (Lezar and van der Walt 2023), and emotional intelligence. Emotional intelligence has been linked to work engagement and positive psychological functioning among nurses, supporting workplace flourishing (Aqtam et al. 2025) and, in high‐intensity settings such as NICUs, to clinical judgment, adaptive coping, and sustained positive work experiences (Ayed 2025).

Leadership styles—including empowering (Peethambaran and Naim 2023), authentic (Kleynhans et al. 2022; Rautenbach 2015), transformational (Maio et al. 2023), and servant leadership (Giolito et al. 2021)—have also been associated with WF.

A related concept, thriving at work, further complements the understanding of positive functioning at work, describing a psychological state of vitality and learning (Jackson 2022). Among nurses, thriving is associated with supportive relationships, positive environments, meaningful work, and learning opportunities, whereas workload, emotional strain, and ethical challenges pose barriers (Satchell and Jacobs 2024; Jackson 2022).

Despite its importance, WF research remains limited, particularly in healthcare (Fabricio et al. 2022; Peethambaran and Naim 2025). Existing studies in healthcare have linked WF to lifestyle factors and sick leave (Mascaro et al. 2022), leadership and psychological resources (Chevalier et al. 2021), workplace interactions (Persson et al. 2021), social environment (Bossard et al. 2024), and value congruence (Cevallos et al. 2023). Furthermore, evidence from Middle Eastern settings, shows that the work environment significantly influences nurses' professional quality of life, highlighting organizational conditions as both facilitators and barriers to WF in high‐intensity settings (Batran et al. 2025). It also identifies notable knowledge gaps, emphasizing the need for targeted educational interventions and providing regional baseline data to improve care practices (Ayed et al. 2025).

Although previous research in healthcare has primarily focused on hospital‐based and high‐intensity settings, WF in occupational health contexts remains underexplored. Given their emphasis on prevention, work ability, and long‐term well‐being, examining WF in these settings is needed to broaden understanding across healthcare contexts.

## 3 The Study

### 3.1 Aim and Research Question

This study aims to describe nurses' experiences of workplace flourishing in healthcare. The research question was: What are nurses' experiences of emotional, psychological, and social well‐being dimensions of workplace flourishing in healthcare?

## 4 Methods

### 4.1 Study Design

This is a qualitative descriptive design using content analysis to explore a phenomenon that is incompletely understood in its natural context (Kyngäs et al. 2020). The Reporting Qualitative Research (COREQ) checklist (Table S1) was used to ensure comprehensive and transparent reporting (Tong et al. 2007).

### 4.2 Theoretical Framework

The theoretical framework guiding this study is based on the concept of flourishing, as proposed by Agenor et al. (2017), and the Flourishing at Work framework (Rautenbach 2015) (Figure 1).

**Figure 1:**
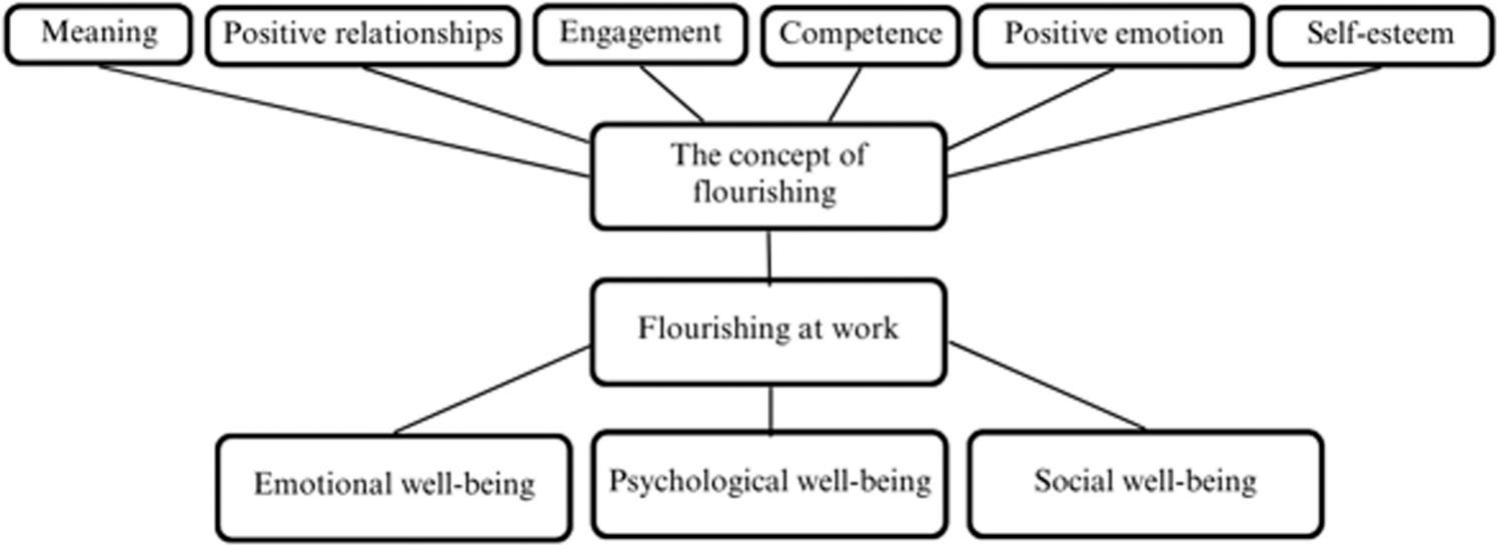
The theoretical framework guiding the study based on and adapted from flourishing concept analysis by Agenor et al. (2017) and the Flourishing at Work framework (Rautenbach 2015). (Authors' own work).

### 4.3 Sampling and Recruitment

Empirical data were collected from nurses (*n* = 16) working in a private occupational healthcare organization operating nationally across Finland. The private occupational health service context was selected to offer a novel perspective on WF in a prevention‐oriented healthcare setting.

The organization provides occupational health services for knowledge‐intensive work, including mental well‐being support and workplace development, and offers a continuum of mental health care from low‐threshold counseling to therapeutic interventions. It was selected due to its pioneering role in developing mental well‐being services and its interest in gaining a deeper understanding of WF within a high‐performing team. Team performance was assessed using internal metrics evaluating meaningfulness at work, quality of working life, work well‐being, and employees' likelihood of recommending the employer.

The study focused on this specific team, with nurses selected through convenience sampling (Polit and Beck 2021). Participants were recruited via an email invitation distributed by the team supervisor, and all volunteers were included in the study. Inclusion criteria required current employment within the team. Of the 19 nurses invited, 3 could not be reached, resulting in 16 participants, including the supervisor with a nursing background.

The selected team was considered an information‐rich case, as WF was expected to be particularly evident, making it suitable for in‐depth exploration. The sample size was deemed adequate for qualitative analysis, providing sufficient information power to address the research aim, given the specific context, relative homogeneity of participants, and richness of the data (Malterud et al. 2016).

### 4.4 Data Collection

Data were collected through semi‐structured individual interviews between January and April 2023. The interview guide covered three main themes—emotional, psychological, and social well‐being—based on Rautenbach (2015) Flourishing at Work framework. The interview questions were developed under these themes (Table 1).

**Table 1:** A semi‐structured interview guide.

Interview themes and questions
Theme 1. Emotional well‐being
What kind of emotions do you experience at work?
How would you describe your general mood and job satisfaction?
To what extent do you feel that your job aligns with your ideal work?
Theme 2. Psychological well‐being
How do you perceive your autonomy and ability to influence your work?
How would you describe your self‐esteem and its impact on your work?
How do you assess your professional competence and learning opportunities?
To what extent do you perceive your work role as clearly defined?
To what extent do you find your work meaningful?
How do you experience your commitment to work and relationships within your work community?
Theme 3. Social well‐being
How would you describe the workplace climate and your sense of belonging?
How do you experience the leadership of your team?

Authors' own work.

The semi‐structured interview guide was pre‐tested with two participating nurses, and no changes were required. The pre‐test interviews were included in the final data. Individual interviews were conducted remotely via Microsoft Teams by one of two researchers (female MSc students, JA, TM) following a standardized protocol, recorded with permission, and attended only by the participant and interviewer. Transcription yielded 297 A4 pages (12‐point Times New Roman, 1.15 line spacing). Interviews lasted 40–122 min (average 64.5 min), totaling 17 h and 12 min. Transcripts were not returned to participants; however, participants could provide additional comments or questions at the end of each interview. Background information (Table 2) was collected via a Webropol survey using established questions to enhance reliability.

**Table 2:** Participants' characteristics (*n* = 16).

Variable	*f*
Gender	
Female	14
Male	2
Highest educational level	
College degree	2
Bachelor's degree	11
Master's degree	3
Professional status	
Nurse	15
Nurse team supervisor	1
	Mean
Age (years)	37.8^a^
Work experience (years)	
In the current work	2.0^b^
In healthcare	9.5^c^

Authors' own work.

^a^ SD: 6.1, min–max: 31.0–49.0.

^b^ SD: 1.2, min–max: 0.08–4.7.

^c^ SD: 5.1, min–max: 2.3–23.8.

### 4.5 Data Analysis

Data were analyzed using deductive–inductive content analysis (Kyngäs et al. 2020), enabling the use of the FAWS framework (Rautenbach 2015) to explore experiences in a healthcare context. The FAWS framework guided the analysis by providing the three overarching dimensions—emotional, psychological, and social well‐being—while categories and sub‐categories were developed inductively from the data.

Transcription was conducted and checked against audio recordings by two researchers (JA, TM). First, two researchers (JA, TM) read the transcripts several times to gain an overall understanding of the data. A meaning unit was selected as the unit of analysis (Kyngäs et al. 2020), and meanings relevant to the research question were coded (*n* = 1906) using NVivo (R1). Coding was conducted collaboratively by two researchers (JA, TM), with ongoing discussion throughout the process and discrepancies resolved through consensus.

After the coding was complete, the deductive method was applied using the FAWS framework as an unstructured analysis matrix, in which the open codes were categorized into emotional, psychological, and social well‐being dimensions. This deductive phase provided an overarching structure for the analysis. Once all codes had been placed within these three main categories, the inductive phase began. This phase focused on grouping the codes based on similarities and differences within each main category, leading to the formation of sub‐categories and categories through abstraction, thereby generating new insights into how WF was experienced in this specific context. A total of 31 sub‐categories and 10 categories were identified. The grouping and abstraction were conducted collaboratively by the two researchers (JA, TM), with ongoing comparison of interpretations to ensure consistency.

Disagreements between researchers (JA, TM) were resolved through iterative discussions, including within the research team, until consensus was reached. The coding tree was developed and refined throughout the analysis (Table S2).

### 4.6 Ethical Considerations

Study permission was granted by the participating organization, which also evaluated the study's ethical aspects. The Ethics committee of Human Sciences of the University of Oulu has considered the ethical principles of the study and, based on Finnish law, ethical review was not required (File S1), as the study did not involve patients or minors and posed no direct or indirect physical or mental harm to participants (World Medical Association 2024). Participation was voluntary. Before the interviews, participants received oral and written information about the study, its aims, and data protection, and provided electronic written consent. Personal data were removed from the transcripts to ensure anonymity, in accordance with national guidelines on responsible research conduct (The Finnish Advisory Board on Research Integrity 2023).

### 4.7 Trustworthiness and Reflexivity

Trustworthiness was evaluated based on credibility, dependability, confirmability, authenticity, and transferability (Kyngäs et al. 2020). Credibility was supported by transparent reporting, appropriate participant selection, and data saturation. Saturation was defined as the point at which no new categories emerged, and additional interviews yielded little novel information (Guest et al. 2006; Nelson 2017). The determination of saturation was based on ongoing data analysis and jointly assessed by two researchers (JA, TM), who reached consensus through iterative discussion. Saturation was reached after 14 interviews, which may also reflect the relatively homogeneous nature of the participants. All scheduled interviews were nevertheless completed. Member checking was not feasible.

Credibility was further enhanced by pilot testing the interview guide, ensuring clarity and relevance. Potential power dynamics related to including a team supervisor were mitigated through confidential individual interviews, which enabled participants to express their views freely.

Dependability was strengthened by providing tables and examples of the analysis process, while confirmability was ensured through detailed category descriptions and collaborative analysis by two researchers (JA, TM), with repeated review by the research team. The absence of field notes may have limited the data's contextual richness; however, verbatim transcripts were used to support analytic rigor. Authenticity was enhanced by systematically using participants' direct quotations (Table S3).

Transferability was supported by clearly defined selection criteria and descriptions of participant backgrounds. As the study focused on WF within a specific private occupational health context, the findings are not intended to represent nursing experiences across all clinical settings; the relatively well‐functioning organizational context should be considered when assessing the transferability of findings.

The interviewers had nursing backgrounds but no prior relationship with participants, reducing potential bias and power imbalance. Two researchers were involved in data collection and analysis, and both continuously reflected and critically assessed their professional backgrounds and the potential influence of their own perspectives on the research process.

## 5 Findings

The nurses' experiences of WF are described in three main categories: emotional well‐being, psychological well‐being, and social well‐being (Figure 2; Table 3).

**Figure 2:**
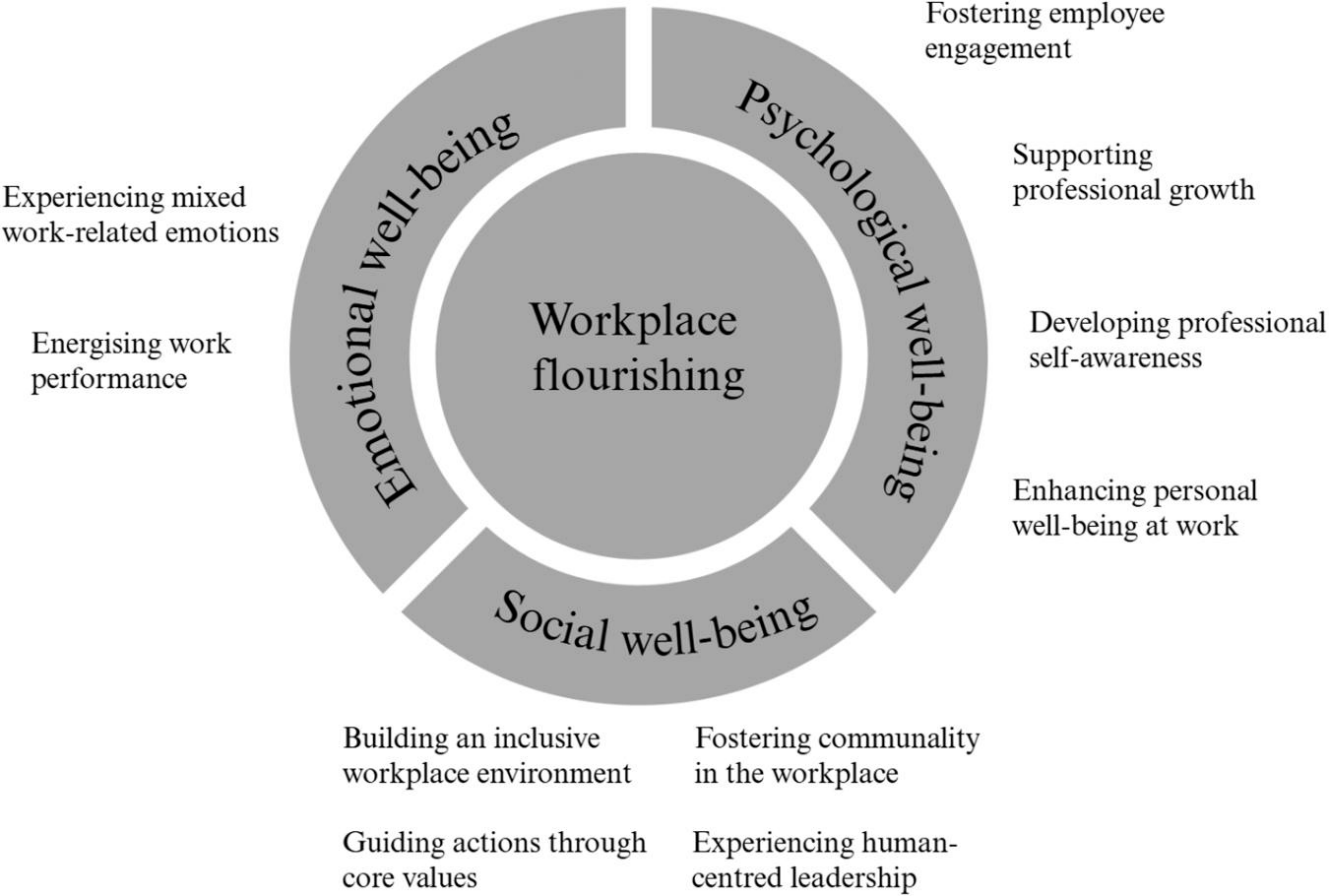
Main categories and categories of nurses’ experiences of workplace flourishing in healthcare. The three main categories represent the core dimensions of workplace flourishing, while the surrounding categories illustrate the related experiences and contextual conditions identified in the data.

**Table 3:** Nurses’ experiences of workplace flourishing in healthcare based on interview data.

Main categories (*n* = 3)	Categories (*n* = 10)	Sub‐categories (*n* = 31)
Emotional well‐being	Experiencing mixed work‐related emotions	Feeling passionate about work Experiencing work as burdensome
	Energizing work performance	Feeling thriving at work Reaching flow at work Achieving a strong sense of energy at work
Psychological well‐being	Fostering employee engagement	Committing to the employer and the work community
		Supporting employee job satisfaction through employer actions Promoting well‐being in society as an employer
	Supporting professional growth	Understanding one's skills at work Sharing expertise within the workplace Identifying learning opportunities at work Fostering a strong desire for the workplace development
	Developing professional self‐awareness	Understanding the significance of self‐esteem in work
		Having a clear understanding of one's role within the workplace
	Enhancing personal well‐being at work	Experiencing good opportunities to influence work within the workplace Finding meaning in work
		Achieving a balance between work and leisure
Social well‐being	Building an inclusive workplace environment	Safe workplace atmosphere Fostering a human‐centered organizational culture Demonstrating a positive attitude towards diversity in the workplace
		Enhancing employee acknowledgment in the workplace
	Guiding actions through core values	Valuing the commendable values of the workplace Guiding one's actions with a strong work ethic
	Fostering communality in the workplace	Experiencing a sense of belonging among members of the workplace
		Building positive relationships within the workplace Receiving strong support from the work community
		Experiencing positive multi‐disciplinary collaboration in the workplace
	Experiencing human‐centered leadership	Perceiving the supervisor as approachable Experiencing the supervisor's empowering approach The coaching approach of the supervisor Having a positive impact on the well‐being of workplace members by the supervisor

Authors’ own work.

### 5.1 Emotional Well‐Being

The first main category, emotional well‐being, included two categories: experiencing mixed work‐related emotions and energizing work performance. Nurses described *experiencing mixed work‐related emotions*, including feeling passionate about their work and feeling burdened by their work. Nurses expressed a passion for their work and emphasized the supportive and trusting atmosphere within their work community. Feeling passionate about work was also supported by the fact that nurses felt their job closely aligned with their ideals, including the variety of tasks, meaningful interactions with people, and opportunities to influence their work. However, nurses also acknowledged the need for better tools and processes to support their passion for working. Experiencing work as burdensome, the nurses identified challenging emotions, such as frustration over inadequate societal resources and a sense of inadequacy from heavy workloads.

Passion (at work) for me probably relates to that, yes, to that meaningfulness, so when I feel that something has been significant, then it also generates in me those, like, those feelings of passion.

—Nurse 4

The nurses described *energizing work performance* as encompassing feeling thriving at work, reaching flow at work, and achieving a strong sense of energy at work. WF was evident in how the nurses were feeling thriving at work, as they expressed satisfaction with both their work and job roles, highlighting their generally upbeat mood in the workplace. Nurses particularly emphasized the importance of the work community in their experience of thriving, and they shared feelings of success, enthusiasm, and joy with their colleagues. According to the nurses, maintaining an appropriate work pace and a good level of alertness helped them to reach a state of flow at work. Flow was experienced as a loss of time awareness, enthusiasm, and deep immersion in the work. WF was reflected in the emphasis on the positive impact of the work community in achieving a strong sense of energy. Face‐to‐face interactions were particularly felt to be energizing, and colleagues were seen as a source of energy, especially during challenging moments.

That energy (at work) is influenced by your well‐being, your endurance, and your personal life, but even during tougher times, you can draw energy from the team.

—Nurse 9

### 5.2 Psychological Well‐Being

The second main category, psychological well‐being, included the following four categories: fostering employee engagement, supporting professional growth, developing professional self‐awareness, and enhancing personal well‐being at work. According to the nurses, *fostering employee engagement* enhances WF by employees committing to the employer and the work community, supporting employee job satisfaction through employer actions, and promoting well‐being in society as an employer. Committing to the employer and the work community was described as resulting from a good work community, the employer, and the work itself. Supporting employee job satisfaction through employer actions was reflected in good leadership, successful recruitment, and sufficient resources. Promoting well‐being in society as an employer was described as an important organizational aim, with nurses perceiving that their employer actively influences societal issues through its services.

I would say that I am highly committed to my work, probably because there has also been a commitment to me; in a way, I get something in return*.*

—Nurse 16

WF was reflected in *supporting professional growth*, including understanding one's skills at work, sharing expertise within the workplace, identifying learning opportunities, and fostering a strong desire for workplace development. Nurses' experiences of WF showed that understanding one's skills at work was important. The nurses felt that their expertise and job‐related skills were at a substantial or sufficient level, and that their work experience further boosted their confidence. Nurses also noted that the fast pace and time pressure affected their ability to manage their responsibilities. Sharing expertise within the workplace was highly valued by the nurses. Knowledge was openly shared, and team members' specialized skills were actively used in training others. Identifying learning opportunities at work, nurses saw their job as a continuous learning process. While they appreciated the available opportunities, they also wished for more training and development. The workplace supported learning through time, encouragement, onboarding, and peer mentoring. Fostering a strong desire for workplace development, the nurses felt the employer was continuously improving operations with employee input, listening to suggestions, and allowing collective decision‐making. Although nurses also felt that personal development opportunities could be improved.

We share like very openly and a lot, we share all kinds of things, which also makes it possible to like adopt others' good practices into one's practice.

—Nurse 4

The nurses expressed that *developing professional self‐awareness* involves understanding the significance of self‐esteem in the workplace and having a clear understanding of one's role within it. The nurses felt that self‐esteem influenced their work, for example, by lowering the threshold for asking for help and affecting how they expressed their opinions and needs. They described their self‐esteem and professional self‐esteem as generally good. Nurses also described their professional self‐esteem as having been strengthened through their current work. The nurses considered having a clear understanding of their role within the workplace as important for WF, although they experienced some ambiguity or desired more clarity. Role divisions between professional groups were also evident.

I feel that my professional self‐esteem is strong, and I have received much reinforcement for it in my current workplace; training each other and the expansion of my role have strengthened it further.

—Nurse 14

According to the nurses, *enhancing personal well‐being at work* supported WF as it included experiencing good opportunities to influence work within the workplace, finding meaning in work, and achieving a balance between work and leisure. The ability to influence one's work, such as its content, working hours, and role, was seen as a key aspect of workplace opportunities. Finding meaning in work was considered important, as nurses perceived their roles as enabling them to make a difference in the world, one person at a time. At the same time, nurses felt their impact was primarily limited to improving individuals' health. Meaningfulness in work often stemmed from helping clients and receiving positive feedback from them or colleagues, as well as from the support of the work community. Achieving a balance between work and leisure was considered essential for promoting WF among nurses. The employer was recognized for supporting work‐life balance through various initiatives, including encouraging hobbies, considering personal stress, and providing flexibility in workload and shift scheduling. Nurses also reported that remote work positively affected their work‐life balance.

The employer gives excellent support, like towards your private life, like they always take into account what kind of load I have in my personal life, and like how I could maybe ease things at work during that time, so that I can keep my head together. And then when things get better again, I can also cope better at work.

—Nurse 11

### 5.3 Social Well‐Being

The third main category, social well‐being, included the following four categories: building an inclusive workplace environment, guiding actions through core values, fostering communality in the workplace, and experiencing human‐centered leadership. The nurses perceived that *building an inclusive workplace environment* was essential for WF, as it required a safe workplace atmosphere, fostering a human‐centered organizational culture, demonstrating a positive attitude towards diversity, and enhancing employee acknowledgment in the workplace. In particular, the safe workplace atmosphere was described as positive, supportive, and open, making it easy to share thoughts and opinions. This safe workplace atmosphere was seen as a facilitator for addressing complex issues and engaging in open discussions. It also encompassed a culture of trust among colleagues, including the ability to share confidential matters, as well as trust in employees, as reflected in leadership.

Furthermore, the nurses observed that the absence of hierarchical structures was a critical element in fostering a human‐centered organizational culture, which contributed to WF by creating a sense of togetherness and warmth across professional boundaries. Demonstrating a positive attitude towards diversity within the workplace was reflected in recognizing and appreciating the differences among team members. Allowing individuals to be themselves was evident, as nurses felt the workplace encouraged everyone to be their true selves. Team members were described as accepting and humane towards each other, contributing to a safe environment where it was possible to express, for example, ignorance and uncertainty. Enhancing employee acknowledgment in the workplace was perceived as a means of ensuring inclusion and consideration in social situations, with nurses feeling recognized, heard, and seen.

Perhaps the most important aspect for me is like the idea of growing together, or something like that, where you do not have to be somehow ‘ready’ or perfect; you can bring yourself as you are, along with your uncertainties and reflections, as well as your successes.

—Nurse 13

According to the nurses, *guiding actions through core values* consisted of valuing the commendable values of the workplace and guiding one's actions with a strong work ethic. Valuing the commendable values of the workplace was considered significant and reflected in the organization's daily practices, contributing to WF. The nurses felt they received honest responses from the employer when addressing problems and believed that individuals were treated with dignity and respect. Additionally, they emphasized the importance of aligning their values with those of the workplace. Guided by a strong work ethic, the nurses viewed it as their duty to deliver the highest standard of care.

I follow a professional ethics where I always say that if I could not take care of my mother in this matter with a clear conscience, then I would not take care of anyone else either.

—Nurse 16

*Fostering communality in the workplace* consisted of experiencing a sense of belonging among members of the workplace, building positive relationships within the workplace, receiving strong support from the work community, and experiencing positive multi‐disciplinary collaboration in the workplace. Experiencing a sense of belonging among workplace members was evident in unity across professional boundaries, with the team perceived as a cohesive unit fostering an environment conducive to WF. Additionally, the company of colleagues evoked positive emotions, as nurses felt they had warm and positive relationships within the workplace. Receiving strong support from the work community was also important for WF, and the nurses felt that they received it from the entire work community. The nurses experienced positive multi‐disciplinary collaboration in the workplace. In particular, the low hierarchy in the workplace was perceived as supporting this collaboration, further contributing to WF.

It is that kind of selfless support, and it is something that everyone wants to give equally, in my opinion.

—Nurse 2

*Experiencing human‐centered leadership* in WF was highlighted as important, including perceiving the supervisor as approachable, experiencing the supervisor's empowering and coaching approach, and perceiving a positive impact on the well‐being of workplace members. Perceiving the supervisor as approachable, nurses described the supervisor as easily accessible, trusted, and an equal member of the team. The supervisor's empowering approach was reflected in nurses' perceptions of leadership, emphasizing soft values such as encouragement and acknowledgment of individual needs. The supervisor's coaching approach was described as both encouraging autonomy and supportive, fostering an environment that promotes WF. Having a positive impact on the well‐being of workplace members, the supervisor also made the nurses feel that they genuinely cared about the team members' well‐being, which supported their WF.

(Manager's name) plays a significant role as a supervisor, as their clear priority is the well‐being of our team.

—Nurse 10

## 6 Discussion

This study provides novel insights into nurses' experiences of WF in healthcare, organized into three main categories based on Rautenbach (2015) Flourishing at Work framework. Regarding emotional well‐being, our results strongly highlighted the positive impact of the work community on WF and its role in fostering energizing work performance. Nurses emphasized the importance of support from colleagues, gaining energy during challenging moments, and sharing successes, enthusiasm, and joy. This aligns with A'yuninnisa et al.'s (2024) study, which noted the positive influence of the work community on WF. In our study, nurses commonly experienced positive emotions at work, consistent with previous studies (Agenor et al. 2017). Muñiz‐Velázquez et al. (2022) similarly found that flourishing employees experience greater happiness at work, supporting job satisfaction and workplace interactions. While our findings emphasize the positive role of collegial support and work community role in fostering energizing, they may under‐represent the ways in which organizational pressures or less supportive team dynamics can limit workplace flourishing in the nursing context.

Findings on psychological well‐being showed that WF was reflected in nurses' engagement with their work, particularly regarding the work community and the employer. Previous studies have shown that WF reduces employees' intentions to leave (A'yuninnisa et al. 2024). Our study also highlighted the role of sufficient employer‐provided resources in supporting strong work engagement, consistent with studies showing that job resources promote WF (A'yuninnisa et al. 2024; Fabricio et al. 2022). The study among NICU nurses has further linked work engagement to emotional intelligence, highlighting the role of individual psychological resources in sustaining engagement in demanding clinical environments (Aqtam et al. 2025), thereby further supporting WF through sustained work engagement. Our findings indicate that nurses' experiences of workplace flourishing are fundamentally shaped by organizational and relational conditions, rather than being solely attributable to individual engagement.

Our findings highlighted the importance of enhancing personal well‐being at work, including opportunities to influence work, finding meaning in work, and achieving work–life balance. Meaningfulness was closely linked to nurses' well‐being and shaped by both individual and community factors. Previous research has associated meaningful work with professional competence and coping with work demands, particularly through emotional intelligence and adaptive strategies (Ayed 2025), and identified it as central to WF (A'yuninnisa et al. 2024; Rautenbach 2015; Satchell and Jacobs 2024). Our study further highlights the need to actively support shared and sustained meaningfulness within the work community, which may have been under‐recognized.

In our study, work–life balance was important for WF, consistent with earlier studies (A'yuninnisa et al. 2024; Chevalier et al. 2021; Mascaro et al. 2022). Nurses emphasized the employer's actions to support work–life balance and the alignment of work with personal life, such as enabling remote work. This differs from Rautenbach (2015), who found that flexible work arrangements were not associated with WF. These inconsistencies may reflect contextual differences in organizational practices and professional roles, suggesting that the impact of work–life balance on WF is shaped by specific work environments. Our findings indicate that nurses' opportunities to influence their work play a central role in WF, aligning with Persson et al. (2021). Our results showed that nurses experienced autonomy and flexibility in their work, aligning with some studies linking autonomy to WF (A'yuninnisa et al. 2024; Jackson 2022; Persson et al. 2021). Additionally, our findings highlight the importance of employee involvement in workplace development, in line with Persson et al. (2021).

Regarding social well‐being, our results suggested that building an inclusive workplace environment promotes WF. Our findings showed that a safe workplace atmosphere was a crucial foundation for WF. This finding aligns with Peethambaran and Naim (2025), who emphasize the importance of psychological safety in enabling employees to express concerns and act authentically. Furthermore, our results are consistent with previous research highlighting the role of the work environment in shaping nurses' professional well‐being, with organizational conditions acting as key facilitators or barriers to WF (Batran et al. 2025). Our findings on guiding actions through core values highlighted the alignment between organizational and individual values, which has previously been noted as positively associated with WF (A'yuninnisa et al. 2024; Cevallos et al. 2023). In our study, a strong work ethic among nurses also emerged as a factor supporting WF, a finding less emphasized in earlier research. This finding suggests that a strong work ethic may function as an internal resource that enhances nurses' sense of purpose and supports WF.

According to our study, fostering workplace communality supported WF and nurses' strong sense of belonging to the work community, as well as positive workplace relationships. Previous studies have also indicated that social relationships and a sense of belonging contribute to WF (Keyes 2002; Mascaro et al. 2022; Satchell and Jacobs 2024). In addition, our results emphasized the importance of strong support from the work community, aligning with studies showing that organizational support contributes to WF (A'yuninnisa et al. 2024; Bossard et al. 2024; Jackson 2022). WF was also evident in organizational support for multidisciplinary collaboration, which was perceived as highly functional and facilitated by a low hierarchy. Although multidisciplinary collaboration is widely studied in healthcare, its link to WF has received limited attention, and its effectiveness may depend on clear structures and leadership to prevent role ambiguity.

Our results demonstrated the supervisor's significance in supporting autonomy and involving nurses in decision‐making. Leadership was characterized by coaching, empowerment, and encouragement, consistent with previous studies linking these factors to WF (Granville‐Chapman et al. 2024; Peethambaran and Naim 2023). The supervisor was also responsive to nurses' individual needs. Previous studies have also found that recognizing subordinates as individuals enhances flourishing, particularly in demanding environments (Maio et al. 2023). Importantly, our study highlighted the essential role of the entire work community in promoting WF in the nursing context, consistent with findings by Granville‐Chapman et al. (2024). While predominantly positive nurses' experiences were found in our study, some negative and challenging aspects were also identified, including feelings of workload burden, role ambiguity, and a need for greater role clarity. Additionally, some nurses perceived their impact as limited to improving individual patients' health. These findings complement the FAWS framework by demonstrating that, alongside supportive elements, challenges persist that may shape nurses' experiences of WF.

The theoretical contribution of this study lies in extending and considering the applicability of the Flourishing at Work framework in nursing. While the framework provided a useful lens, the findings suggest it does not fully capture the context‐specific nature of flourishing in nursing. Notably, social and psychological well‐being were particularly emphasized, whereas emotional well‐being was less prominently reflected. The categories constructed within these dimensions provide novel, practically applicable insights into how flourishing manifests in nursing.

### 6.1 Limitations

This study has some limitations. The homogeneity of the sample, with all participants recruited from a single private occupational healthcare organization in Finland and the majority being female, may limit the transferability of the findings to other contexts. The selection of a high‐performing team may also have influenced the findings by emphasizing positive experiences of workplace flourishing and reflecting a relatively well‐functioning organizational context.

All interviews were conducted via Microsoft Teams, which may have affected the interview process, although participants were familiar with the platform due to remote work. The qualitative interview design may have introduced social desirability bias, as participants may have portrayed their work experiences more favorably. Recruitment via the team supervisor may have introduced perceived coercion, potentially affecting voluntary participation and the representativeness of the sample. The findings should be interpreted with caution due to potential selection bias associated with convenience sampling. The absence of member checking may be considered a limitation, as the findings were not validated with participants.

### 6.2 Recommendations for Further Research

Future research should examine WF across diverse healthcare contexts and professional groups to deepen understanding of its determinants and manifestations. Studies examining the relationships among WF, patient care outcomes, and productivity could yield valuable insights into the broader impact of WF on healthcare systems. In addition, there is a need for intervention studies that develop and evaluate educational and action‐based interventions, such as leadership training, peer support programmes, and organizational practices, to support and enhance WF.

## 7 Conclusions

The results showed that a positive work community strongly supported all dimensions of WF, underscoring the fundamentally social nature of WF. Human‐centered leadership—expressed through coaching, empowerment, and genuine interpersonal support—was viewed as essential for fostering WF, suggesting a need for targeted leadership training. Nurses' WF was also shaped by personal factors, including core values, meaningful work experiences, and growth opportunities, while peer support could further strengthen social connection and belonging. Additionally, having influence over one's work and organizational support for work–life balance emerged as important contributors to WF, highlighting the need to integrate these elements into organizational practices. This could include organizational policies that promote flexible scheduling and other practices that enable nurses to balance professional responsibilities with personal life.

## 8 Implications for the Field

These findings can help healthcare organizations strengthen awareness of WF and its benefits. By promoting human‐centered leadership, cultivating a positive and inclusive work community, and aligning organizational practices with individual values, organizations may enhance nurses' well‐being, support retention, and improve overall performance. Nurse supervisors also play a key role by recognizing individual needs and supporting the holistic well‐being of their teams. At the organizational and healthcare system levels, healthcare administrators could embed principles of workplace flourishing into organizational and system‐level strategic planning, leadership development, and workforce well‐being policies. The key role of organizational culture in sustaining workplace flourishing indicates that this is not solely an individual‐level phenomenon, but one that requires systemic support.

## Author Contributions

Conception, conceptualization, and design of the manuscript: Annanolli Josefiina, Mäenpää Tiia, Kuha Suvi and Kanste Outi. Acquisition of data: Annanolli Josefiina and Mäenpää Tiia. Analysis and interpretation of data: Annanolli Josefiina and Mäenpää Tiia. Writing the manuscript: Annanolli Josefiina, Mäenpää Tiia, Kuha Suvi and Kanste Outi. Commenting on the manuscript: Annanolli Josefiina, Mäenpää Tiia, Kuha Suvi and Kanste Outi.

## Ethics Statement

Permission was obtained from the target organization to conduct the research. According to Finnish legislation, this study did not require approval from the Research Ethics Committee, as the interviews did not involve patients or minors and did not interfere with their physical or mental well‐being.

## Conflicts of Interest

The authors declare no conflicts of interest.

## Supporting information


Supporting File 1

Supporting File 2

Supporting File 3

Supporting File 4

## Data Availability

The authors have nothing to report.
