# Supplementary material for: Nurses' Experiences of Workplace Flourishing in Healthcare: A Qualitative Study
Source: Res Nurs Health. 2026 Aug 1;49(6):790–800. doi: 10.1002/nur.70109 (PMC13729727; doi:10.1002/nur.70109)
Supplement: Supplementary file 3 [file NUR-49-790-s002.docx]

Supplementary Table 3. Nurses’ original expressions by category.

| Category | Original expression |
| --- | --- |
| Experiencing mixed work-related emotions | ‘Perhaps the positive feeling comes from being able to help people in many ways and of different ages, too. That sense of making a difference brings positive emotions and a feeling of accomplishment, especially when someone gives feedback.’–Nurse 15.  ‘The strongest positive emotions arise either when I’m with my colleagues in our comfortable workplace, or when I talk about my work outside of work, when I reflect on it more deeply and think or speak about what I like about my job and why I enjoy it.’–Nurse 4.  ‘Lately, I’ve been feeling quite overwhelmed. Of course, a lot of that strain comes from other areas of life as well, so this job hasn’t really been the cause; it has actually been more of a positive factor overall.’–Nurse 10. |
| Energising work performance | ‘For me, energy at work is completely linked to how well and how much I’ve slept. That really affects how energetic I feel. Of course, if all the basic things in life are in balance, it naturally boosts your energy. But if you’re overwhelmed with worries and things on your mind, it drains your capacity to work, and then you don’t necessarily feel very energetic.’–Nurse 3.  ’I want to say that when I’m physically back at the workplace, I really feel that sense of community, the culture, the collegiality, how should I put it, also the energy; it all comes through much more strongly for me.’–Nurse 4.  I have enough energy for my work. The physical workload isn’t too heavy, but you do notice that even sitting can be tiring and doing this kind of mental work as well. But then again, as I mentioned earlier, by planning and structuring my work myself, I can adjust the workload so that I still have energy left for my free time and other interesting things. Overall, I would say my energy levels are good.  ’–Nurse 16. |
| Fostering employee engagement | ’I do feel committed to this job. It’s been a good workplace, one where I enjoy working, so I haven’t had the feeling that I should start looking for other job opportunities.’–Nurse 1.  ‘The reason I’m still at (employer’s name) and haven’t looked for other jobs is simply because I like this workplace so much, both the whole organisation and especially the work community within our team.’–Nurse 13.  ‘...this organisation genuinely tries to act in the best interest of us employees.’–Nurse 14. |
| Supporting professional growth | ‘There are opportunities for learning at work. We have regular, scheduled team meetings, and if someone sees a training opportunity outside the company that would clearly benefit both the employee and the organisation, they’re usually given the chance to attend. That said, we’ve also discussed that there could be more training overall; it’s something that really needs more investment, but resource constraints have been a challenge.’–Nurse 9.  ‘In my opinion, we make good use of the expertise that exists within the organisation, and that leads to new learning.’–Nurse 4.  ‘I would say the resources for managing challenging tasks are good. What stands out in particular is the support from colleagues and the wider work community. If your own expertise isn’t enough, you can always put your head together with others to ask questions or figure things out. You can always ask for help, and the situation gets resolved one way or another.’–Nurse 1. |
| Developing professional self-awareness | ‘Well, regarding work, I feel that I have good self-esteem, because I know that I know a lot of detailed information related to our job. And, well, on the other hand, over the years my self-esteem has also grown in the sense that I also know what I don’t know, and I can admit that openly and ask for help if I don’t know something.’–Nurse 7.  ‘I think my self-esteem is pretty good, like I dare to bring up those (stress factors), and I’m able to talk about them with my supervisor or bring up my own insecurities within the work community.’–Nurse 13.  ‘I think the (work role) is apparent, there’s nothing unclear, well, at least for me it’s very clear what belongs to me, like I said, I delegate quite readily, what falls under my responsibilities and what belongs to someone else, so yeah.’–Nurse 10. |
| Recognising the broader significance of work | ‘Well, we do have a societal impact in that sense, since we’re part of occupational health care, and thousands of people have a contract with us. So the things that are handled through us are potentially something that don’t have to be taken care of in the public healthcare system.’–Nurse 14.  ‘I see my work as important and meaningful, and that was a big, big reason why I decided to change careers. I was looking for a new sense of meaning in my work, and I’ve found it.’–Nurse 4.  ‘I do feel that I'm doing work that has a purpose, especially when I get appreciation from the client’s side or from colleagues, from anyone really, it clearly gives the feeling that I’ve been able to help someone, and yes, that definitely feels meaningful.’–Nurse 1. |
| Enhancing personal well-being at work | For me at least it has appeared that there is at the moment a constant desire to develop, like like opinions are asked. It's asked how we could do better. It's listened, listened really well to all those wishes and in my opinion there is reacted really quickly, so that it doesn't remain as just empty words, that it's not just tried at some point but that then actual concrete changes are made even with a fast schedule, if the situation so requires.’–Nurse 2.  ‘One can really influence those work shifts.’–Nurse 12.  ‘(Organisation name) has excellent means to reconcile, like, work life and civilian life or private life, and if in private life there are situations, challenges, needs, then (organisation name) really does everything... so that the person can reconcile those, and they meet halfway, are flexible.’–Nurse 4. |
| Building an inclusive workplace environment | ‘We have a safe work environment and the work community is such that where you can bring out your own thoughts and can influence it like or like a safe environment where you can bring out those own thoughts and that is also encouraged, whether they are positive or also negative feelings, that they are not or thoughts that they are not tried to be suppressed in any way, but rather it is supported that you can say your own opinion honestly.’–Nurse 1.  ‘This is really easygoing, there isn’t like that kind of hierarchy, maybe I’d say in that, and then when we at times meet somewhere with the whole company’s staff, like, then it is really, really jovial.’–Nurse 3.  ‘So like now I’m talking about diversity, so yes I have that kind of feeling that we relate to it well.’–Nurse 3. |
| Guiding actions through  core values | ‘I feel that, and it is probably an essential resource, that my values match in this team really well. That people are treated well, and others are valued and respected, is important to me; we are fair, kind, and accepting. And then I feel that in this team, like also that kind of difference, different values are accepted.’–Nurse 6.  ‘Well that (alignment between the work community and personal values) was also one reason, when I applied here for work, like I wouldn’t have come to work if they weren’t like in line with the values, so so yeah, the values are side by side with my own way of thinking and like, that I can with a straight back say, that that like offer and give the best possible help like for that situation.’–Nurse 16.  ‘I try like until the very end to still do my job well, because I feel that it is, it is like in a way both an honour and it belongs to that professional ethics and to that, that you handle the matter as well as you can.’–Nurse 15. |
| Building communality  in the workplace | ‘There are like very warm and confidential relationships, like I was just thinking that surely one could, if needed, talk about anything and everything like that, knowing that it is confidential.’–Nurse 16.  ‘That support is easily gotten (from the work community) and, like in a completely different way than in any other workplace, it is really pushed on you. And I’ve also heard the same from others, that people are quite grateful for the support they have received.’–Nurse 11.  ‘We at (organisation name) don’t have white coats on the doctor like they say and hierarchy, so we are equal when treating people, so that increases the sense of community.’–Nurse 5. |
| Fostering mutual respect in the workplace | ‘Our team is definitely the best, most functional, funniest team I have worked in, and actually, I have to bring in a word now here, because it should have somehow been turned into a feeling also already at the start – trust. It's an essential word in our team and in the whole (organization name), yes, so we have strong trust like both in each other’s professionalism, competence, that trust shows in leadership, that there is trust that people do their own their own work and do it well, and and then then that trust is also strongly reflected in our team spirit.’– Nurse 4.  ‘Here one becomes seen and heard, noticed and in all ways like probably some of those basic social human needs are fulfilled, so to speak, also at work, that is quite, maybe it’s not so common.’– Nurse 3.  ‘Actually, that kind of feeling of being met, seen, heard is actually really important, if I think about what I experience in my own team and in my own organisation.’– Nurse 4. |
| Experiencing human-centered leadership | ’(Supervisor) tries to help in everything, comes themself to work if someone is suddenly missing or if sudden help is needed.’– Nurse 8.  ‘They have been really like the kind of supervisor I haven’t had before, like they have been really, really close, it’s really easy to talk to them about things.’– Nurse 12.  ‘A very people-oriented, very empowering kind of leadership, like maintaining the person she is, so like and then that kind of encouraging and also like that she really encourages people a lot, like to strengthen their own competence.’–Nurse 5. |

(Authors’ own work)
