# Supplementary material for: Nurses' Experiences of Workplace Flourishing in Healthcare: A Qualitative Study
Source: Res Nurs Health. 2026 Aug 1;49(6):790–800. doi: 10.1002/nur.70109 (PMC13729727; doi:10.1002/nur.70109)
Supplement: Supplementary file 4 [file NUR-49-790-s003.docx]

Supplementary Table 2. Example of the deductive–inductive content analysis process within the category fostering communality in the workplace under the dimension of social well-being.

| Open codes | Sub-categories | Category |
| --- | --- | --- |
| The experience that the work community is almost on the same level as family and friends  Feels that their team is exceptionally tightly bonded  Feels that their team is truly a close-knit group, even though they work remotely from all over Finland  Feels that there is a genuine sense of togetherness in the work community | Experiencing a sense of belonging among members of the workplace | Fostering communality  in the workplace |
| Feels that the entire organisation is one united group  Feels that collegiality is one of the main driving forces  Feels a sense of belonging with their coworkers, even though everyone is very different |  |  |
| Feels that the company has chosen open-minded and positivity-bringing people  Feels that all relationships within the work community are positive  A feeling of joy when meeting coworkers  Through the work community, has found people who are now also friends  Feels that all relationships within the work community are warm and trusting  Feels that they have never been part of a work community with such a strong sense that everyone is a good person | Building positive relationships within the workplace |  |
| Feels that within the work community, support and help are always available, regardless of professional group  Feels that within the work community, people genuinely want to help each other selflessly, which feels new and pleasant  Feels that they receive help within the work community even when they have not realised how to ask for it  Support and understanding are provided by coworkers and supervisors in challenging situations  Feels that the support from the work community is extremely strong and positive  Feels that they manage challenging work tasks well with the support provided by the work community  Feels that they can always call their assigned 'mentor' at work if they need help or support | Receiving strong support from the work community |  |
| Feels that working with the multidisciplinary team has been incredibly easy  Feels that they can easily consult the multidisciplinary team whenever they do not know something  Feels that the low hierarchy between professional groups enhances the sense of community  Feels that it is easy to consult the doctors  A low threshold to contact anyone in the organisation, which has a positive impact on the work  Feels that continuous learning comes through multidisciplinary collaboration, as they can consult experts from other fields and learn in the process | Experiencing positive multi-disciplinary collaboration in the workplace |  |

(Authors’ own work)
